# A Maternal-Effect Toxin Affects Epithelial Differentiation and Tissue Mechanics in *Caenorhabditis elegans*

**DOI:** 10.3389/fcell.2021.743496

**Published:** 2021-10-14

**Authors:** Christina Lehmann, Christian Pohl

**Affiliations:** Buchmann Institute for Molecular Life Sciences and Institute of Biochemistry II, Medical Faculty, Goethe University, Frankfurt, Germany

**Keywords:** Discs large, tissue mechanics, apical junction, *C. elegans*, morphogenesis, epidermis, selfish genetic element

## Abstract

Selfish genetic elements that act as post-segregation distorters cause lethality in non-carrier individuals after fertilization. Two post-segregation distorters have been previously identified in *Caenorhabditis elegans*, the *peel-1*/*zeel-1* and the *sup-35*/*pha-1* elements. These elements seem to act as modification-rescue systems, also called toxin/antidote pairs. Here we show that the maternal-effect toxin/zygotic antidote pair *sup-35*/*pha-1* is required for proper expression of apical junction (AJ) components in epithelia and that *sup-35* toxicity increases when pathways that establish and maintain basal epithelial characteristics, *die-1*, *elt-1*, *lin-26*, and *vab-10*, are compromised. We demonstrate that *pha-1(e2123)* embryos, which lack the antidote, are defective in epidermal morphogenesis and frequently fail to elongate. Moreover, seam cells are frequently misshaped and mispositioned and cell bond tension is reduced in *pha-1(e2123)* embryos, suggesting altered tissue material properties in the epidermis. Several aspects of this phenotype can also be induced in wild-type embryos by exerting mechanical stress through uniaxial loading. Seam cell shape, tissue mechanics, and elongation can be restored in *pha-1(e2123)* embryos if expression of the AJ molecule DLG-1/Discs large is reduced. Thus, our experiments suggest that maternal-effect toxicity disrupts proper development of the epidermis which involves distinct transcriptional regulators and AJ components.

## Introduction

In search for embryonic lethal mutations, developmental geneticists have uncovered numerous organ master regulators and terminal differentiation factors in the nematode *Caenorhabditis elegans*, the arthropod *Drosophila melanogaster*, or the chordate *Danio rerio*. PHA-1, a presumably organ-specific differentiation factor for the *C. elegans* feeding organ, the pharynx, was identified in the early 1990s (Schnabel and Schnabel, 1990). Work from the Schnabel Laboratory showed that *pha-1* is an essential gene in the *C. elegans* isolate N2 as it was required for the terminal morphogenesis of presumably all five pharyngeal cell types, namely, muscle, marginal, epithelial, gland, and nerve cells during the time window of mid-embryogenesis (Schnabel and Schnabel, 1990). Subsequent work by the Fay Laboratory revealed that *pha-1* temperature sensitive alleles show synthetic lethality at the permissive temperature when the retinoblastoma pathway (*efl-1*/E2F, *lin-35*/Rb) or a specific ubiquitination pathway (*ubc-18*/UBCH7, *ari-1*/HHARI) are inhibited (Fay et al., 2004; Mani and Fay, 2009).

Besides these synthetic lethal interactions, three suppressors of the embryonic lethal *pha-1* loss-of-function phenotype were obtained, namely, *sup-35, –36*, and –*37* (Schnabel et al., 1991). Cloning and further characterization of these suppressors showed that they might associate with each other, that they all seem able to bind microtubules directly or indirectly, and that they might undergo regulated translocation from the cytoplasm to the nucleus during organogenesis (Mani and Fay, 2009; Fay et al., 2012; Polley et al., 2014). Specifically, *sup-35* encodes a C2H2 zinc finger protein with a claimed similarity to members of the Regulators of Microtubule DyNamics family (RMDN/Fam82; Mani and Fay, 2009). The *sup-36* encodes a divergent Skp1-like protein that is most likely part of Skp1-Cullin-F-box (SCF) ubiquitin ligase complexes, can associate with the microtubule-binding protein PTL-1/tau, and can regulate SUP-35 localization (Polley et al., 2014). *sup-37* encodes a nuclear C2H2 zinc finger protein that does not seem to have orthologs outside nematoda but is essential for coordinating rhythmic pharyngeal pumping and ovulation independently of *pha-1* (Fay et al., 2012). Moreover, while SUP-35 and –36 seem to be negatively regulated by ubiquitination through UBC-18/ARI-1, this does not apply for SUP-37 (Fay et al., 2012). Based on extensive genetic interaction analyses and transgenes, it has been argued that the most likely function of SUP-35/36/37 lies in transcriptional regulation, which is spatiotemporally regulated by the developmental stage-specific and constitutively cytoplasmic factor PHA-1 (Fay et al., 2012).

Recent work from the Kruglyak Laboratory (Ben-David et al., 2017) now allows to re-interpret the afore-mentioned data. By comparing the *C. elegans* isolate N2 (Bristol) with highly divergent *C. elegans* isolates, it became clear that their genomes neither contain *pha-1* nor *sup-35* coding sequences and that presence of PHA-1 and SUP-35 in N2 is due to a derived haplotype through gene inversion (Ben-David et al., 2017). Based on this data, rather than acting as a pharyngeal differentiation regulator, PHA-1 can be considered a zygotic antidote or repair factor of a post-segregation distorter-type selfish genetic element (Ben-David et al., 2017). In this element, SUP-35 acts as the maternally supplied toxin which seems to exert a cell-type specific toxicity that can be antagonized by zygotically expressed PHA-1. This interpretation is consistent with previous data that already showed *pha-1* loss-of-function phenotypes when SUP-35 is overexpressed in the background of wild-type (WT) *pha-1* (Mani and Fay, 2009). Moreover, N2 lacking both PHA-1 and SUP-35 are viable (Fay et al., 2012). Thus, *pha-1* and its suppressors *sup-35*, *–36*, and *–37* are not conserved between *C. elegans* isolates. Yet, *sup-35* nevertheless seems to target conserved pathway/s. If *sup-35/pha-1* indeed acts as a maternal-effect selfish genetic element in the form of a post-segregation distorter, SUP-35 must exert a highly developmental stage-specific toxicity. Since SUP-35 is provided maternally and PHA-1 is only expressed after the onset of gastrulation (Ben-David et al., 2017), basic cellular functions cannot be the target of SUP-35 or a SUP-35/-36/-37 complex. Moreover, in *pha-1* loss-of-function backgrounds, cells do not undergo cell death or stop proliferating but fail to execute specific aspects of morphogenesis. Thus, the *pha-1* loss-of-function suppressors potentially target a developmentally regulated program rather than general cellular functions. Notably, the Fay Laboratory has previously described additional roles of PHA-1, namely, roles in epithelial cells of the pharynx and in the epidermis for morphogenesis, in the excretory system for osmoregulation, in the somatic gonad for normal ovulation and fertility, and in some intestinal cells for viability (Kuzmanov et al., 2014). These results suggested that PHA-1 might be a regulator of epithelial morphogenesis.

**FIGURE 1:**
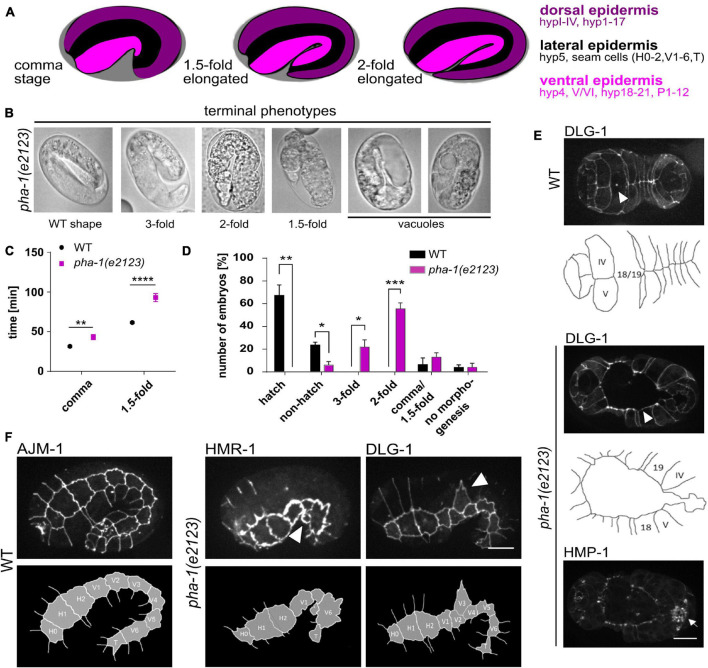
*pha-1* mutations affect epidermal morphogenesis. **(A)** Schematic of *C. elegans* elongation stages and epidermis organization. **(B)** Brightfield images of *pha-1(e2123)* embryos that arrested at different developmental stages (1. 5–, 2–, and 3-fold). **(C)** Quantification of elongation time (from lima-bean to the 1.5-fold stage) in WT and *pha-1(e2123)* embryos (with SEM, *n* = 12/13). **(D)** Normalized quantification of embryonic arrest in WT and *pha-1(e2123)* animals mounted with 20 μm beads (with SEM, *n* ≥ 90). **(E)** Maximum projections and corresponding schemes of ventral enclosure in WT and *pha-1(e2123)* embryos expressing DLG-1::dsRed and HMP-1::GFP. Arrow heads point at cell boundaries of leading cells found at the ventral midline. In *pha-1(e2123)* embryos ventral enclosure fails in rare cases. Mutant embryos however properly position sensillae (arrow). **(F)** Maximum projections and corresponding schemes of seam cell localization in WT and *pha-1(e2123)* embryos expressing AJM-1::GFP, HMR-1::GFP and DLG-1::dsRed. Arrow heads indicate mis-localization and misshaping of single seam cells. **(D,E)** Scale bars, 10 μm. T-test: *p < 0.05; **p < 0.01; ***p < 0.001; and ****p < 0.0001.

The epidermis of *C. elegans* is a one-layered epithelium that encases the entire animal (Chisholm and Hardin, 2005) to protect it from extrinsic factors (Pujol et al., 2008). Besides being a barrier, during mid-embryogenesis, it regulates morphogenetic processes like embryonic elongation (Priess and Hirsh, 1986; Williams-Masson et al., 1997; Costa et al., 1998), cell and axon guidance (Hedgecock et al., 1990), it secretes basement membrane material that separates it from underlying body wall muscles (Francis and Waterston, 1991), and eliminates apoptotic cells and synapses *via* phagocytosis (Robertson and Thomson, 1982; Chung et al., 2000). Importantly, epidermal cells provide the force that drives body elongation (Priess and Hirsh, 1986; Shelton et al., 1999; Wissmann et al., 1999; Piekny et al., 2003; Gally et al., 2009; Vuong-Brender et al., 2016; Shaye and Soto, 2021). The different stages of embryonic elongation in *C. elegans* have been coined with names that reflect the overall shape or elongation progress (Figure 1A), while the invariantly numbered and positioned epidermal cells also have a specific nomenclature (also see Figure 1A). To drive elongation, epidermal cells need to be properly fated. The master regulator for the specification of epidermal cells is the GATA transcription factor ELT-1, which is expressed in all epidermal cells from the onset of gastrulation (Page et al., 1997). ELT-1, in turn, drives expression of ELT-3 (Gilleard et al., 1999), the zinc-finger transcription factor LIN-26 (Labouesse et al., 1994), and the nuclear hormone receptor NHR-25 (Gissendanner and Sluder, 2000) that are required for additional aspects of epidermal differentiation and morphogenesis. The expression of *lin-26* starts at the 100-cell stage in all epidermal precursor cells and is maintained throughout adulthood to lock epithelial fate in these cells (Labouesse et al., 1994, 1996). Initiation and maintenance of epidermal fate through LIN-26 relies on its role in driving expressions of apical junction (AJ) components and apicobasal polarity determinants. However, for internal organs with epithelial character like the mouth, pharynx, and intestine, the developmental regulator PHA-4 takes over this role and represses *lin-26*, presumably by binding to its promoter (Gaudet and Mango, 2002; Landmann et al., 2004).

Unlike vertebrates and insects, where AJs can be divided into two distinct regions, only a single continuous structure has been observed in *C. elegans* (Knust and Bossinger, 2002). Nevertheless, the *C. elegans* AJ can still be sub-divided in two complexes, an apical cadherin- α-/β-catenin complex (CCC) and a more basally located DLG-1/AJM-1 complex (DAC) (Köppen et al., 2001; McMahon et al., 2001). The CCC in *C. elegans* comprises homologs of the classical CCC of vertebrates and flies, HMR-1 (E-cadherin), HMP-1 (α-catenin), HMP-2 (β-catenin), and JAC-1 (p120-catenin) (Costa et al., 1998; Pettitt et al., 2003). Just like CCCs in other organisms, the CCC of *C. elegans* mediates cell–cell adhesion which plays an essential role in embryogenesis as inhibition of contact formation during morphogenesis leads to developmental arrest (Costa et al., 1998; Raich et al., 1999). The DAC contains the membrane-associated guanylate cyclase (MAGUK) scaffold protein DLG-1 (Discs large in Drosophila) and the nematode-specific AJ component AJM-1. This complex, together with LET-413 (Scribble in Drosophila), serves as a polarization cue for the distribution of apical and basolateral factors and thus divides the epithelial membrane into distinct functional zones (similar to tight junctions in mammals). DLG-1 inhibits an apical localization of LET-413 whereas LET-413 restricts the DAC and apical factors to the apical surface (Legouis et al., 2000; Bossinger et al., 2001; Firestein and Rongo, 2001; Köppen et al., 2001; McMahon et al., 2001). This represents another special feature of the AJ in *C. elegans*, as Discs large and Scribble in Drosophila co-localize (Bilder et al., 2000). Furthermore, DLG-1 and AJM-1 show an interdependent localization *in vivo*, and DLG-1 is required to anchor AJM-1 to the cell boundary (Firestein and Rongo, 2001; Köppen et al., 2001; McMahon et al., 2001; Lockwood et al., 2008). Another phenotype of *dlg-1* and *ajm-1* null mutants is posterior vacuolization, indicating that they also control paracellular gating (Firestein and Rongo, 2001; Köppen et al., 2001; McMahon et al., 2001).

Here, we investigate the mechanism of *sup-35* toxicity in N2 by using a highly penetrant, temperature-sensitive loss-of-function mutation of *pha-1*, *pha-1(e2123)*. We demonstrate that rather than affecting pharyngeal tissue specifically, toxicity manifests in defects in epidermal morphogenesis by altering expression of AJ components, specifically, the DAC components AJM-1 and DLG-1 during mid to late elongation. However, we also find that expression of the CCC component HMR-1 in the pharynx is significantly altered throughout the entire elongation process. These data suggest a tissue-specific effect on spatiotemporal regulation of epithelial gene expression programs. Accordingly, by performing a targeted RNAi screen, we demonstrate that several of the main pathways responsible for epithelial differentiation and morphogenesis, *lin-26*, *let-1*, and *die-1* exacerbate the *pha-1(e2123)* phenotype at the permissive temperature. In addition, we find that *pha-1(e2123)* embryos show reduced cell bond tension during elongation. Furthermore, we uncover that by laser cutting dorsoventral cell–cell contacts, a significant reversal of elongation can be induced in WT embryos, most likely due to the previously described stiffness anisotropy in the dorsal epidermis (Vuong-Brender et al., 2017) and force anisotropy (recently reviewed in Carvalho and Broday, 2020). This elastic response is completely lost in *pha-1(e2123)* embryos and can be recovered by reduced expression of the AJ component DLG-1 or by creating mechanical stress through uniaxial loading, pointing to overall altered tissue biomechanics. Accordingly, we show that *dlg-1* RNAi can rescue embryonic elongation in *pha-1(e2123)* embryos. We provide evidence that these phenomena, altered spatiotemporal expression of AJ components, altered cell bond, and tissue mechanics are most likely resulting in the terminal phenotypes described previously.

## Materials and Methods

### Strains

Strains were obtained from the Caenorhabditis Genetics Center (CGC) {strains: FT250 [*unc-119(ed3) III*; xnIs96]; GE24 [*pha-1(e2123)* III]; JJ1473 [*unc-119(ed3)* III; zuIs45 V]; ML916 [mcIs40; lin-15B&lin-15A(n765) X]; N2; SU93 [(jcIs1 IV); SU265 (jcIs17)]} or have been generated by crossing (strains: CHP59 by crossing SU265 with GE24; CHP61 by crossing SU93 with GE24; CHP62 by crossing FT250 with GE24 and CHP72 by crossing ML916 with GE24). Strains were maintained under standard conditions at 20 or 15°C (temperature sensitive mutants).

### RNAi

RNAi was performed by feeding as described earlier (Dutta et al., 2019) using clones from commercially available libraries^1^ or prepared from gDNA. All experiments, with exception of *pat-*2 and *vab-10* in GE24, were performed using L1 staged larva. For *pat-2* and *vab-10*, RNAi L4 worms were used since use of L1 larva led to severe egg laying defects (*pat-2*) or lethality in young L4s (*vab-10*). Worms were maintained at 19°C [weak penetrance of *pha-1 (e2123)* mutant phenotype, ∼80% of embryos hatch], or 25°C [high penetrance of *pha-1 (e2123)* mutant phenotype].

### Microscopy

Embryos were dissected from gravid hermaphrodites, mounted in 2.5 μl of an M9 buffer suspension containing either 25, 20, or 15 μm poly-styrene microspheres (Polyscience, Warrington, PA, United States), and sealed between two coverslips (Roth, Karlsruhe, Germany) with vaseline. Details of compression under these conditions have been published previously (Singh et al., 2019). Microscopy was performed with a VisiScope spinning disk confocal microscope system (Visitron Systems, Puchheim, Germany) based on a Leica DMI6000B inverted microscope, a Yokogawa CSU X1 scan head, and a Hamamatsu ImagEM EM-CCD as described earlier (Dutta et al., 2015). All acquisitions were performed at 21–23°C using a Leica HC PL APO 63x/1.4-0.6 oil objective.

### Laser Ablation

For laser ablation experiments, the microscope was additionally equipped with a 2D-VisiFRAP DC System with an integrated 355 nm pulsed UV laser (Visitron Systems, Puchheim, Germany; see also Singh et al., 2019). The FRAP cycle was performed after two intervals pre-ablation (intervals = 250 ms) with a FRAP time per pixel of 2–2.5 ms. The cell boundary between V3 and V4 of embryos with varying length (≤55 μm; H0 – V5) was ablated and recoiling/body retraction was measured using ImageJ. Embryos were harvested from gravid mothers maintained at either 25 or 19°C and data is represented as an average of 5–7 embryos with SEM.

### Signal Intensity Measurements

Fluorescence intensities of CeAJ-components were measured with ImageJ using a small spherical region of interest in the tissue of interest during varying developmental stages (lima-bean, comma, and 1.5-fold). Embryos were always measured with the same orientation, only the objective-facing side of left-right mounted embryos (mounted with 20 μm beads) was used to measure fluorescence intensities to exclude measuring far-side signals. Embryos that were mounted dorsoventrally (using 25 μm beads) were not used for measurements. Signals were measured at three different regions on cell–cell contacts within the tissue and means of measured values were background subtracted placing the ROI outside the embryo. Data is represented as an average of 4–6 embryos with SEM. Signal intensities for AJM-1::GFP following *die-1* RNAi and for ABD_VAB–10_::mCherry were measured with ImageJ using line tools and the Plot Profile function.

### Viability Assay

L1 larva of WT or *pha-1(e2123)* were put on NGM-agar plates seeded with *Escherichia coli* OP50 or an RNAi clone and maintained at 19°C until adulthood. Progeny was scored for embryonic arrest, larval arrest, and developing animals until 48 h after mothers started to lay obviously aberrant eggs. Developing progeny was removed from plates reaching L3 stage.

### Developmental Timing and Arrest

Gastrulating embryos (maximum of 5 per mount) were harvested from gravid mothers maintained at 25 or 19°C and mounted under standard conditions using 20 or 15 μm poly-styrene microspheres (Polyscience, Warrington, PA, United States). To analyze developmental arrest, mounts were kept at 19 or 25°C for 24 h and examined for their developmental stage under the microscope. Elongation time was determined through confocal fluorescence microscopy by measuring the time required for development from lima-bean (amphid pores start to move anterior) to 1.5-fold stage.

### Twitching Analysis

Wild-type and *pha-1(e2123)* embryos of varying lengths (≤55 μm; H0 – V5) were mounted and imaged as described before (time intervals = 300 ms). Twitching was measured with ImageJ using line tools and the Kymograph function.

## Results

### *pha-1* Is Required for Proper Epithelial Morphogenesis

While examining *pha-1(e2123)* embryos to study how defective pharynx development impinges on sensory organ morphogenesis, we found that head enclosure was strongly delayed in mutant embryos. Further examination of this phenotype revealed strong differences in AJM-1::GFP, a component of the basally located DAC, relative to WT embryos, pointing to a role for *pha-1* in embryonic morphogenesis as previously suggested (Kuzmanov et al., 2014). In addition, we frequently observed epidermal deformations and vacuoles in *pha-1(e2123)* embryos (Figure 1B). Moreover, we found that besides head enclosure, the entire process of body elongation was significantly delayed in *pha-1(e2123)* embryos (from 61 ± 5 min to complete body elongation from lima-bean to 1.5-fold stage to 92 ± 18 min) (Figure 1C), 13 ± 7% of *pha-1(e2123)* embryos arrested between comma and 1.5-fold stage of elongation, 55 ± 9% at twofold and 22 ± 11% at the threefold stage, while only 5% of the *pha-1(e2123)* embryos examined showed WT morphology (Figure 1D). Less often, we found *pha-1(e2123)* embryos with ventral enclosure defects (Figure 1E) and with loss, mis-localization, or deformation of seam cells (Figure 1F). Some animals with failed ventral enclosure also displayed a disconnection of the gut from the pharynx primordium. However, failure in ventral enclosure did not impair sensilla positioning even though head enclosure was not always completed. Therefore, we abandoned our initial plan to use *pha-1(e2123)* to study head and anterior nervous system development. Instead, we focused on the function of *pha-1* in epithelial morphogenesis.

### Differential Expression of Apical Junction Components

**FIGURE 2:**
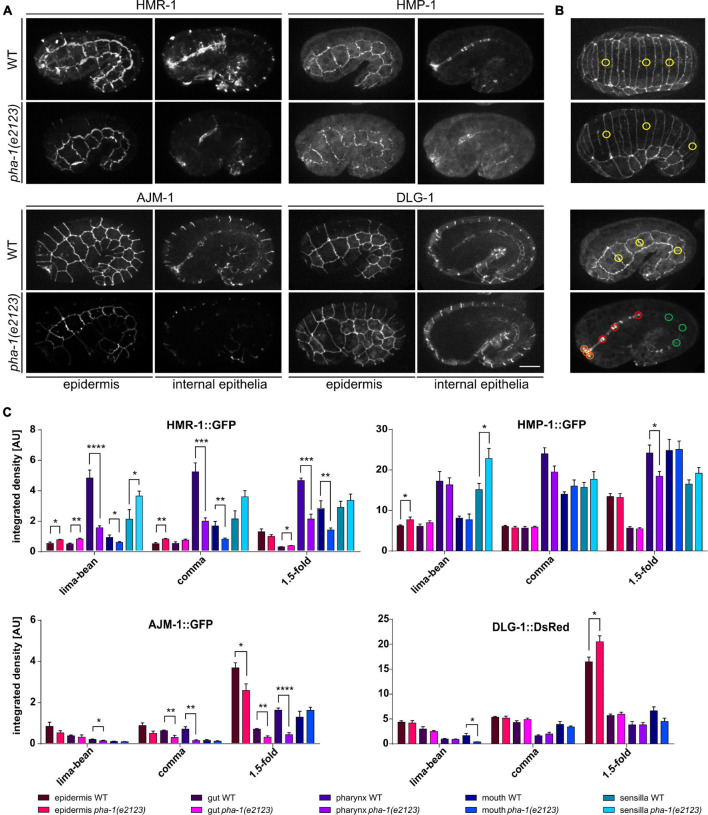
*pha-1(e2123)* embryos show altered expression of CeAJ-components. **(A)** Maximum projections of WT and *pha-1(e2123)* embryos at 1.5-fold stage expressing HMR-1::GFP, HMP-1::GFP, AJM-1::GFP, and DLG-1::dsRed. Shown are the epidermis and internal epithelia (pharynx, gut, and mouth). Scale bar, 10 μm. **(B)** Representative stages for measurements of signal intensities of CeAJ-components in *C. elegans* embryos (from top to bottom: lima-bean, comma, 1.5-fold epidermis, and internal epithelia). Colored circles mark areas used for measurements (yellow, epidermis; green, gut; red, pharynx; and orange, mouth). **(C)** Quantification of signal intensities of HMR-1::GFP, HMP-1:;GFP, AJM-1::GFP, and DLG-1::dsRed in different epithelia (epidermis, gut, pharynx, mouth, and amphid sensillae) of WT and *pha-1(e2123)* embryos (with SEM, *n* ≥ 4; *p < 0.05). Signals were measured during lima-bean, comma, and 1.5-fold stage. T-test: *p < 0.01; ***p < 0.001; and ****p < 0.0001.

Next, we asked, whether the delay in elongation and morphological changes of the epidermis is due to an altered expression of AJ components. We used a variety of strains to compare the expression of HMR-1 (E-cadherin), HMP-1 (α-catenin), AJM-1, and DLG-1 (Discs large) in epidermis, gut, pharynx, mouth, and labial sensilla during different developmental stages (lima-bean, comma, and 1.5-fold) (Figure 2). In WT embryos, expression of HMR-1 is strong in pharynx, mouth, and sensilla (for the latter during the 1.5-fold stage, Figure 2A) and remains so in all developmental stages, while we found a gradual increase for HMP-1, AJM-1, and DLG-1 with increasing elongation (Figures 2B,C). The increase in expression was most intense in the pharynx (in case of HMP-1) and in the epidermis (for AJM-1 and DLG-1) (Figure 2C). Sensilla showed no obvious signal for AJM-1 and DLG-1 (Figure 2A) and, therefore, signal intensities were not measured in this tissue. The expression of AJ components in the gut remained comparatively low for all components tested (Figures 2A,C). The expression pattern was generally altered in mutant embryos (Figure 2C), although the degree of changes in expression varied, strongest changes were observed for HMR-1, downregulation in the pharynx and mouth for all stages, and upregulation inside the epidermis (in early stages), gut, and sensilla. Even though the expression of HMP-1 was largely unchanged, there was a trend-wise upregulation in sensilla and a downregulation within the pharynx. AJM-1 downregulation was most apparent in the mouth, pharynx (starting at comma stage), and epidermis (at the1.5-fold stage). DLG-1 showed no changes in signal intensities except in the epidermis during the 1.5-fold stage, where it became significantly upregulated.

### Screen for Potential *pha-1*-Dependent Regulators in a Sensitized Background

**FIGURE 3:**
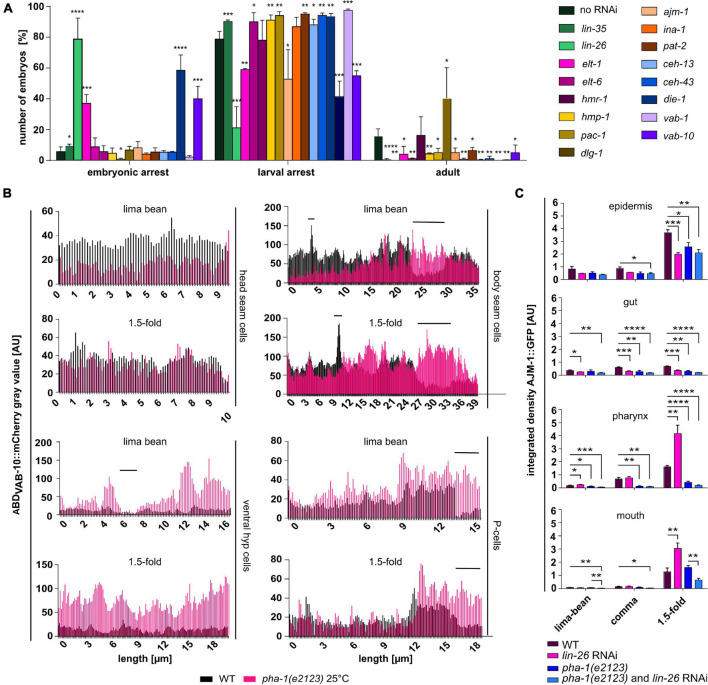
Simultaneous downregulation of PHA-1 and epidermal differentiation- and morphogenesis factors changes viability of *C. elegans* embryos and larvae. **(A)** Viability assay of *pha-1(e2123)* mutants maintained at threshold temperature (19°C) with or without feeding RNAi for epidermal differentiation- or morphogenesis factors. Progeny was scored for embryonic arrest, larval arrest, or full development (adult) (data with SEM, *n* = 3 each; exceptions: no RNAi – *n* = 5; *dlg-1* RNAi – *n* = 9). **(B)** P*lin-26*::ABD_VAB–10_::mCherry expression in head seam cells, body seam cells, ventral hypodermis, and P-cells. Shown are signal intensities of representative WT and *pha-1(e2123)* embryos for lima-bean and 1.5-fold stage. **(C)** Quantification of signal intensities of AJM-1::GFP in different epithelia (epidermis, gut, pharynx, and mouth) of WT and *pha-1(e2123)* embryos with or without *lin-26* feeding RNAi maintained either at the non-permissive temperature of 25°C [*pha-1(e2123)* and *pha-1 (e2123)*/*lin-26* RNAi] or at the threshold temperature of 19°C (WT *lin-26* RNAi) (with SEM, *n* ≥ 4). Signals were measured during lima-bean, comma, and 1.5-fold stage. T-test: *p < 0.05; **p < 0.01; ***p < 0.001; and ****p < 0.0001.

Since *pha-1* seemed to play a role in epidermal development, we performed a screen to analyze combinatorial phenotypes of PHA-1 with other factors that control epidermal differentiation and morphogenesis. Our targeted screen included transcription factors known to directly regulate epithelial patterning (*lin-26, elt-1, elt-6, ceh-13*/HoxB/D, *ceh-43*/DLX1/6, *die-1*; also see section “Introduction”), AJ components or associated/regulatory factors (*hmp-1*/α-catenin, *pac-1*/ARHGAP23, *dlg-1*/Discs large, and *ajm-1*), factors regulating cell–cell adhesion (*hmr-1*/E-cadherin, *ina-1*/integrin α, *pat-2*/integrin α, *vab-1*/EphR, and *vab-10*/plectin), and *lin-35* as a positive control. After a stepwise increase of the permissive temperature (15°C), we identified 19°C as the threshold temperature at which the vast majority (84%) of *pha-1(e2123)* mutants arrested in development (79 ± 5% L1 arrest, 6 ± 3% embryonic arrest). Using this temperature, morphogenesis factors were downregulated using RNAi by feeding. Progeny was scored for arrest during embryogenesis or during larval stages and for adult viability (Figure 3A). Importantly, this screen does not directly identify synthetic interactors since hits from this screen had to be tested in WT backgrounds to judge whether an effect is synergistic, additive, or compensatory. RNAi of all factors but of HMR-1 and AJM-1 significantly increased larval lethality. We also found that downregulation of LIN-26, DIE-1, ELT-1, and VAB-10 in the sensitized background dramatically increased embryonic lethality. Remarkably, we observed that the downregulation of DLG-1 in *pha-1(e2123)* significantly increased viability by almost twofold, which suggests that loss of DLG-1 seems to act in a pathway that can overcome the block in embryonic morphogenesis in *pha-1(e2123)*.

Taken together, our targeted screen suggests that the *sup-35/pha-1* element might indeed play a role in epithelial morphogenesis. Although the results from this screen cannot be directly taken as evidence for synergy or compensation, they nevertheless point to these two testable types of regulation, namely, (1) exacerbation of the *pha-1(e2123)* phenotype if key epithelial transcription factors like DIE-1 or LIN-26 are depleted, and (2) partial rescue of the *pha-1(e2123)* phenotype if the epithelial MAGUK scaffold protein DLG-1 is depleted. To confirm the validity of this interpretation, we focused on these three hits.

### Effects of *pha-1(e2123)* on Epidermal Actin

To further characterize the roles of the *sup-35/pha-1* element in morphogenesis, we analyzed specific aspects of epithelial dynamics. Circumferential actin bundles are located within the epidermis and are key to transmission of forces from seam cells that drive elongation (Priess and Hirsh, 1986; Williams-Masson et al., 1997; Costa et al., 1998; Gally et al., 2009). As *pha-1(e2123)* mutant embryos displayed a significant delay in body elongation, we reasoned that actin dynamics might be altered. We analyzed actin expression in the epidermis by line intensity plots of WT and *pha-1(e2123)* embryos using the transgene *lin-26p*::ABD_VAB–10_::mCherry (Figure 3B, black graphs). Due to the variability of transgene expression, we decided to display representative examples rather than averages of ensembles. For the WT embryo, signal intensity within a certain group of epidermal cells was generally constant, regardless of their location along the anteroposterior axis or progression of elongation. The only exception to this was in P-cells, where posterior cells showed stronger signals than anterior cells. A comparison of signal intensities among different cell types of WT embryos showed that all cell types, except body seam cells (V-cells), showed relatively comparable signal intensities and did not change notably between lima-bean and 1.5-fold stages. In *pha-1(e2123)* embryos, a different pattern was observed (Figure 3B, red graphs). All epidermal cell types (but P-cells) showed changes in signal intensities from lima-bean to 1.5-fold stage. Whereas both seam cell groups showed an increase in signal intensities, the ventral hypodermis showed a decrease. In addition to P-cells, body seam cells displayed higher signal intensities in posteriorly located cells (for body seam cells, this increase was more obvious during lima-bean stage) compared to anterior cells. Hence, despite the variability in mutant embryos, we obtained evidence that altered elongation in *pha-1(e2123)* is in part due to dysregulation of actin in the epidermis.

### AJM-1 Expression and Apical Polarity in *pha-1(e2123)*

Having observed these changes in epidermal actin, we next asked how synthetically lethal interactors of *pha-1(e2123)* alter expression of junction markers. To this end, we analyzed the expression of AJM-1::GFP in different epithelia and at different developmental stages (see Figure 2B for a schematic of measurements) and focused on *lin-26* RNAi first, since we observed the strongest synthetic lethality for this gene in our screen. The *lin-26* RNAi resulted in a decrease of the AJM-1 signal in the epidermis (at 1.5-fold stage) and gut (all stages) but to an upregulation in pharynx and mouth (at 1.5-fold stage) in the WT background (Figure 3C). When comparing *pha-1(e2123)* animals with and without RNAi, significant changes in signal intensities were only observed for the mouth with reduced levels in *pha-1(e2123)* and *lin-26* RNAi embryos (Figure 3C). Thus, although LIN-26 seems to act as a major modulator of AJM-1 expression and of the *pha-1(e2123)* phenotype, it seems to act only as a minor modulator of AJM-1 expression in the *pha-1(e2123)* background.

**FIGURE 4:**
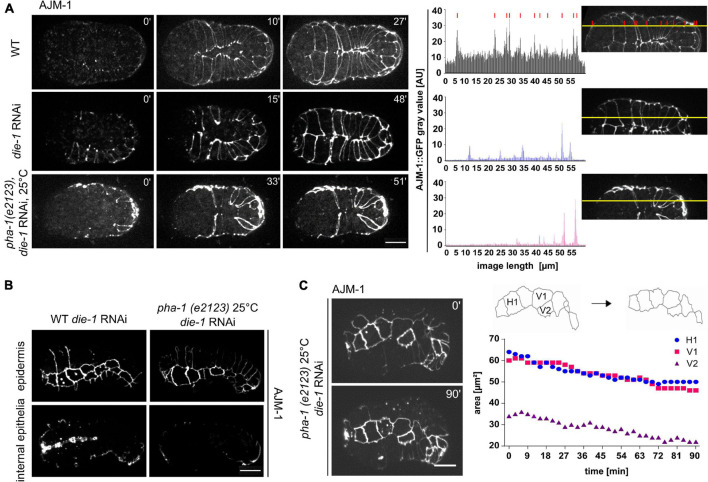
*die-1* RNAi epidermal phenotypes and their exacerbation by *pha-1(e2123)*. **(A)** Left: comparison of ventral enclosure in WT (with or without *die-1* RNAi) and *pha-1(e2123)* embryos. WT embryos exposed to *die-1* RNAi show delayed ventral enclosure due to disorganized migration of contralateral ventral epidermal cells. Note that *pha-1(e2123)* shows unequal expression of AJM-1::GFP in ventral epidermal cells. Right: plot profiles of AJM-1::GFP signals of embryos depicted in the top panel. Yellow lines represent ROIs used for the plot profile. Red lines mark peaks of cell boundary signals. Background signals are indicated through solid black lines in the plot profile. Scale bar, 10 μm. **(B)** Comparison of the impact of *pha-1* and *die-1* on internal and external epithelia. The top pictures show surface (epidermis) signals, bottom pictures show internal (pharynx) signals for AJM-1; scale bar, 10 μm. **(C)** Quantification of changes in cell surface area of single seam cells throughout time following *die-1* RNAi. Left panel: stills of seam cells of *die-1* RNAi embryos. Note that embryos do not elongate, and seam cells show changes in shape. Right panel top: schemes of changes in cell shape of seam cells depicted in left panel. Right panel bottom: quantification of cell surface of single seam cells (H1, V1, and V2) of the embryo shown in the left panel.

Next, we turned to the potential interactor with the second-highest lethality, *die-1*. *die-1* has been named by its loss-of-function phenotype, dorsal intercalation, and elongation defective (Heid et al., 2001). In addition to problems in dorsal intercalation, the original characterization of the *die-1* phenotype also reported problems in ventral epidermal morphogenesis. When ventral epidermal cells migrate toward the ventral midline, they precisely converge with their contralateral partners (Williams-Masson et al., 1997). Consistent with the original characterization (Heid et al., 2001), this precise liaison was disrupted in *die-1* RNAi-treated WT embryos and contralateral partners were slightly displaced along the ventral midline (Figure 4A). This in turn seemed to delay the enclosure process as the time required for full enclosure was longer than in WT embryos. This phenotype was highly penetrant in *die-1* RNAi embryos, and we only infrequently observed this in *pha-1(e2123)* embryos (see above). Unfortunately, we were not able to obtain enough time-lapse recordings to establish a significant effect on synergy on this phenotype. Nevertheless, a closer analysis of the effects of *die-1* RNAi embryos revealed a differential expression pattern of AJM-1 within the epidermal sheet. Cells of the sheet displayed strong reduction and, in the case of *pha-1(e2123)/die-1* RNAi embryos, an almost complete loss of signals (Figure 4A, right) which we never observed for *pha-1(e2123)* embryos alone. In addition to these phenotypes, *pha-1(e2123)/die-1* RNAi embryos showed an almost complete loss of AJM-1 expression for internal epithelia when compared to *die-1* RNAi embryos alone (Figure 4B). Moreover, when further analyzing *pha-1(e2123)/die-1* RNAi embryos, we observed a shrinkage of apical seam cell area to the extent that some seam cells were completely lost from the epithelial layer (Figure 4C). We only observed this in the compound RNAi/mutant embryos and did not observe this phenotype in either *die-1* RNAi only or *pha-1(e2123)* only embryos. These findings indicate that *pha-1(e2123)* exacerbates the problems of expression of AJ factors in *die-1* RNAi and that *pha-1(e2123)/die-1* RNAi embryos might acquire an additional vulnerability concerning maintenance of apical identity.

### Effects of *pha-1(e2123)* on Seam Cell Mechanics

**FIGURE 5:**
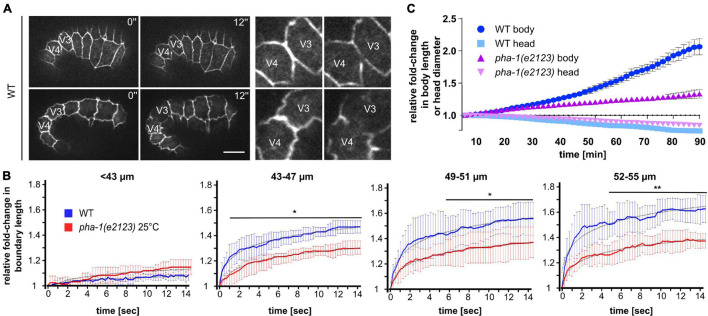
*pha-1(e2123)* mutants exhibit reduced cell bond tension and elongation. **(A)** Maximum projected stills from time-lapse microscopy of laser-ablated WT embryos expressing DLG-1::dsRed at different stages of elongation. Laser ablation was performed at the cell boundary between V3 and V4. Left: total view of embryos; right: detailed view of ablated cell boundaries. Scale bar, 10 μm. **(B)** Normalized quantification of recoil of V3/V4 cell boundaries post-ablation for WT and *pha-1(e2123)* embryos of varying length (≤55 μm, length: anterior side of H0 – posterior side V5), (with SD, *n* = 5 each, start = 1). **(C)** Comparison of changes of head diameter in proportion to embryonic body length over time for WT and *pha-1(e2123)* embryos (with SEM, *n* = 4 each, start length = 1). T-test: *p < 0.05; **p < 0.01.

The above observations led to the question of whether the changes in epithelial morphogenesis in *pha-1(e2123)* embryos were caused by changes in actomyosin-based cortical tension within epidermal cells. We first used laser cutting of cell–cell contacts to monitor forces based on recoil (Colombelli and Solon, 2013). Time-lapse imaging and measuring the recoil of a cell–cell junction immediately after a cut by a laser provides direct insight into the amount of contractile force that was applied to the junction before ablation (initial recoil). To this end, we used a short-pulse (ms) UV laser and cut the cell–cell contact between seam cells V3 and V4 in WT and *pha-1(e2123)* embryos of varying lengths and measured the initial recoil velocities (Figure 5A). We chose this site for ablations since elongation has been shown to proceed by tension increase in lateral epidermal cells and tension release in dorsal and ventral epidermal cells (Gally et al., 2009). We found no obvious recoil in WT embryos shorter than 43 μm that represents embryos younger than 1.3-fold stage (Figure 5B, left). When reaching >43 μm, we saw that with increasing body length, embryos displayed an increase in recoil velocity, indicating an overall increase in tension with progression of elongation (Figure 5B, second to fourth graph). We also observed that the recoil was significantly reduced in *pha-1(e2123)* animals as compared to WT starting at around 43–47 μm of body length. Notably, the initial recoil velocity (first 1 s for 43–47 μm; first 5 s for 49–51 μm; first 4 s for 52–55 μm) did not differ significantly between WT and *pha-1(e2123)*. Significant changes were measured for the steady state at later time points. These changes were also visible as a stronger extension of the boundary length post ablation in WT as compared to *pha-1(e2123)* (Figure 5A).

Elongation is based on a gradual reduction of the diameter of the embryo through contractile force anisotropy within the lateral epidermis and stiffness anisotropy within the dorsoventral epidermis (Vuong-Brender et al., 2017). However, this mechanism is also spatially anisotropic due to the head initially showing little to no reduction whereas the body already decreases in diameter but stops to do so at an earlier time point as the head. Therefore, to corroborate our data on recoil, we compared the ratio of reduction in head diameter to body elongation in WT and *pha-1(e2123)* embryos. Accordingly, we found, as expected, that at the stage where we performed our measurements (1.3- to 1.5-fold embryos), the head of WT embryos initially showed a much less profound decrease in diameter when compared to the strong increase in body length (Figure 5C, light and dark blue). This behavior was similar for the *pha-1(e2123)* mutant, however, while the elongation speed of WT embryos increased with the progression of elongation, the mutant showed no such increase in speed throughout the measurement (Figure 5C, light and dark purple). Hence, we concluded that cell bond tension in the force-generating seam cells is compromised in *pha-1(e2123)* embryos, which manifests in reduced body elongation.

### Effects of *pha-1(e2123)* on Tissue Mechanics

**FIGURE 6:**
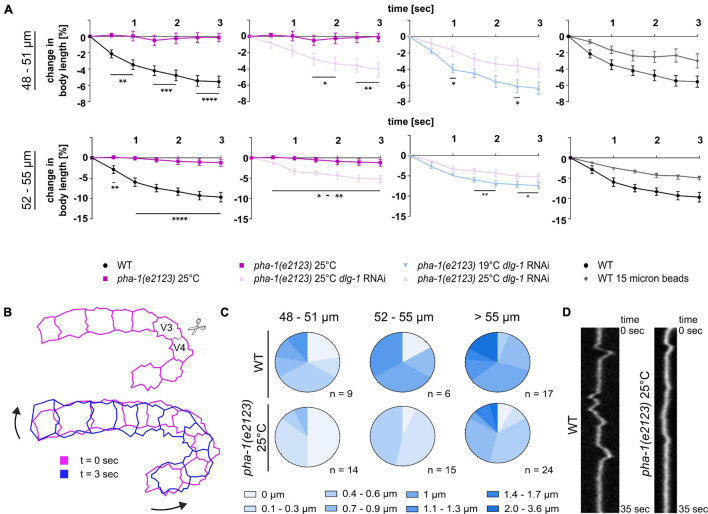
Tissue biomechanics are altered in *pha-1(e2123)* embryos. **(A)** Normalized quantification of body retraction post ablation for WT and *pha-1(e2123)* embryos of varying length (48–55 μm, length: anterior side H0 – posterior side V5) maintained either at the non-permissive temperature of 25°C or at the threshold temperature of 19°C with or without *dlg-1* feeding RNAi. Panel furthest to the right: Comparison of body retraction between WT embryos mounted with 20 or 15 μm microspheres (SEM, *n* = 5–7). **(B)** Scheme of movements observed post laser ablation. Top: Cell boundary within the lateral line of seam cells cut during laser ablations. Bottom: Changes in orientation of seam cells before (*t* = 0 s) and after ablation (*t* = 3 s). Arrows indicate direction of movement of cell sheet. **(C)** Quantification of twitching intensities of WT and *pha-1(e2123)* embryos of varying length (≥48 μm). **(D)** Representative kymograph of cell boundary deflection in WT and *pha-1(e2123)* embryos. T-test: *p < 0.05; **p < 0.01; ***p < 0.001; and ****p < 0.0001.

During the above laser cutting experiments, we also observed that WT embryos displayed a reduction in body length (body retraction), resulting in a straightening of the embryo (Figure 6B). This body retraction, just like the recoil, increased with progression of body elongation and was almost completely absent in *pha-1(e2123)* embryos >1.3-fold stage at restrictive temperature (Figure 6A, left graphs). Since our expression analysis revealed an upregulation of DLG-1 in the epidermis of *pha-1(e2123)* embryos during the 1.5-fold stage (Figure 2C) and based on the finding that increased viability of progeny when DLG-1 was downregulated *via* feeding RNAi (Figure 3A), we tested how the *pha-1(e2123)* body retraction phenotype would be altered by *dlg-1* RNAi. We found that downregulation of DLG-1 led to a partial rescue of the body retraction phenotype at restrictive (25°C) temperature, and to a less strong rescue at the sensitized (19°C) temperature that we had used in our targeted screen (Figure 6A). Hence, these findings indicate that DLG-1 has a strong effect on either cell bond tension in the lateral epidermis and/or on the stiffness of the dorsal epidermis with each effect alone or in combination leading to the observed rescue of elongation and a restoration of tissue mechanical properties.

Since the above effects almost exclusively reflect cell-autonomous epithelial morphogenesis (Vuong-Brender et al., 2017), we also wanted to explore non-autonomous effects that have been characterized as an actin-based viscoplastic lock mechanism ensuring progressive body-axis elongation (Lardennois et al., 2019). Beyond the 1.7-fold of elongation, embryos start to twitch within the egg due to spontaneous muscle activity. Forces generated thereby trigger a mechano-transduction pathway in the epidermis that reinforces elongation (Zhang et al., 2011). Therefore, we analyzed onset and intensity of twitching within wild-type and *pha-1(e2123)* embryos to investigate whether non-autonomous mechanisms of elongation were also affected. We imaged WT and *pha-1(e2123)* embryos with high temporal resolution (250 ms intervals) to follow twitching. We found that with increasing length, the intensity of twitching became stronger (Figures 6C,D) and that WT embryos, when reaching a length of >59 μm, started to turn around their anteroposterior body axis. The *pha-1(e2123)* embryos showed a strong reduction in twitching intensity (Figures 6C,D) and turning of embryos around their anteroposterior body axis was observed the earliest for individuals that had reached a length of 61 μm. This data demonstrates that *pha-1(e2123)* embryos show additional phenotypes that might be related to cell non-autonomous effects.

### Similarities of *pha-1(e2123)* Phenotypes and Extrinsic Mechanical Stress

**FIGURE 7:**
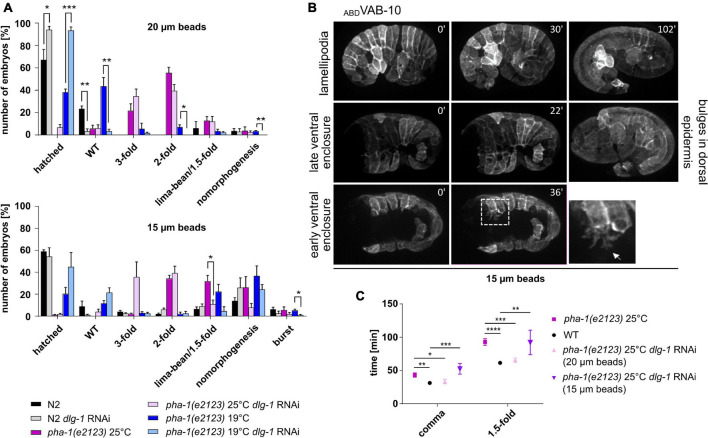
Uniaxial compression partially phenocopies *pha-1(e2123)*. **(A)** Comparison of developmental arrest of WT and *pha-1(e2123)* embryos mounted with 20 or 15 μm microspheres. Embryos were maintained either at the non-permissive temperature of 25°C or at the threshold temperature of 19°C with or without preceding *dlg-1* feeding RNAi (with SEM, *n* ≥ 90). **(B)** Maximum projected stills from time lapse microscopy of embryos expressing *lin-26*p::ABD_*VAB*–10_::mCherry, mounted with 15 μm microspheres. Embryos often exhibit halt of ventral enclosure at any given progression state with ventral cells forming large actin-rich protrusions (arrow in detailed view). In rare cases epidermal cells form large lamellipodia (upper row) or bulges in the head hypodermis (right panel). **(C)** Quantification of elongation time (from lima-bean to 1.5-fold stage) in WT and *pha-1(e2123)* embryos maintained at 25°C and mounted with 20 or 15 μm microspheres with or without preceding *dlg-1* RNAi (with SEM, *n* = 4–8). T-test: *p < 0.05; **p < 0.01; ***p < 0.001; and ****p < 0.0001.

We have previously established a parallel plate assay using different size microbeads to exert forces on embryos and could show that the resulting uniaxial loading can induce strong increases in cortical tension (Singh et al., 2019). Mounting of embryos with 20 μm microspheres leads to a confinement of about 20% and a limited amount of uniaxial loading. A minority of WT embryos (around 2–3% for each condition) showed failure in morphogenesis when mounted with 20 μm microspheres, and around 23% of WT embryos did not manage to hatch. When mounted under these conditions, though, they did not display obvious morphological defects (Figure 7A, top, gray bars). However, for *dlg-1* RNAi-treated embryos, 94% managed to exit the eggshell under these conditions, suggesting that downregulation of DLG-1 seems to somehow balance the external force. Remarkably, the same effect was also observed for *dlg-1* RNAi-treated *pha-1(e2123)* embryos, giving rise to an almost complete rescue at sensitized temperature (Figure 7A, top, blue bars) and a modest rescue at restrictive temperature (Figure 7A, top, purple bars). Next, we were mounting embryos with 15 μm microspheres to increase the mechanical load. The proportion of embryos that failed morphogenesis significantly increased for all conditions (Figure 7A, bottom). Some embryos even burst, which had not been observed for animals mounted with 20 μm microspheres. For *pha-1(e2123)*, we found a trend toward decreased elongation which was not as pronounced for WT. We also tested whether *dlg-1* RNAi could again rescue this developmental arrest and discovered that the rescue effect was abolished in most cases although we found a trend toward improvement for *pha-1(e2123)* embryos at the sensitized temperature (Figure 7A, bottom, blue bars).

To determine the morphogenetic defects that cause a stop in elongation and a subsequent arrest during uniaxial confinement, we imaged embryos mounted with 15 μm microspheres that express the epidermal marker ABD (VAB-10)::mCherry. Ventral enclosure (and subsequently elongation) was often impaired under these mounting conditions, leading to a developmental arrest of the embryo (Figure 7B). In this regard, it made no difference whether ventral enclosure had just begun or had already progressed. Migration of the ventral epidermal sheet was halted at any given stage. Leading cells showed protrusions that were largely arborized and constantly changed in morphology. This applied not only in leading cells but also ventral pocket cells formed such large protrusions (Figure 7B, dashed area in lower row of panels). In cases where ventral enclosure was already terminated, the embryo managed to elongate, and the majority of elongated individuals displayed no obvious alterations in their epidermal phenotypes. Interestingly, in rare cases, some epidermal cells formed lamellipodia that were spreading over or underneath neighboring cells. In other cases, some embryos showed bulges in the head region during late elongation stages (around the 1.5-fold stage) (Figure 7B, middle panels).

Since stronger uniaxial loading abolished or diminished the rescuing effect seen for the downregulation of DLG-1 and embryos showed reduced elongation, we wanted to investigate whether other rescuing effects like the time required for body elongation and body retraction were lost as well under these mounting conditions. We mounted WT and *pha-1(e2123)* embryos with 15 and 20 μm microspheres and quantified the elongation time [with and without *dlg-1* RNAi for *pha-1(e2123)* embryos]. We observed that elongation time could be rescued to WT levels only with 20 μm microspheres and *dlg-1* RNAi (Figure 7C). To better compare this, we also quantified body retraction of WT embryos mounted with 15 μm microspheres and saw that under these conditions, embryos showed a reduced body retraction as compared to WT embryos mounted with 20 μm microspheres (Figure 6A, rightmost panels). This data demonstrates that uniaxial confinement of the embryo partially mimics the effects seen in *pha-1(e2123)* mutants and underlines the notion that the *sup-35/pha-1* element plays a role in controlling mechanical properties of the epidermis through DLG-1.

## Discussion

Observations of presumptive epidermal malformation in *pha-1(e2123)* led to the assumption that PHA-1 might have a broader role in *C. elegans* embryonic development (Kuzmanov et al., 2014). Our experiments demonstrate that PHA-1 seems to be involved in epidermal morphogenesis by attenuating the toxic effect of SUP-35 (Figure 1). To characterize these effects in detail, we provide new quantitative data on the differential spatiotemporal expression of AJ components (Figure 2). In lima-bean to comma stage embryos, junctional components are evenly distributed along cell–cell contacts. In 1.5-fold embryos, HMR-1/E-cadherin and HMP-1/α-catenin show a significant reduction within epidermal cells whereas AJM-1 and DLG-1 remain evenly distributed. Moreover, our measurements revealed a strong increase in AJM-1 and DLG-1 expression in the epidermis which is absent for HMR-1 and HMP-1. On the contrary, in the pharynx primordium, high amounts of HMR-1 and HMP-1 can be found throughout the entire process of foregut development whereas AJM-1 and DLG-1 show higher expression levels only at late stages (during the onset of pharynx elongation). For the epidermis, the transition from lima-bean to 1.5-fold stage represents the time with potentially high mechanical stress (dorsal intercalation, body enclosure, and elongation). This also applies to the pharynx during elongation. The dynamical spatiotemporal changes in junctional components, therefore, most likely reflect mechanical stress leading to junction remodeling as has been characterized in detail before in other systems (for example, see Cowan et al., 1998; Langille, 2001; Huveneers et al., 2012).

We suggest that the rescue of several phenotypes of *pha-1(e2123)* by downregulation of DLG-1 might depend on internal organization of epidermal actin and/or its anchorage, thereby altering the mechanical properties of the tissue. Vuong-Brender et al. (2017) showed that circumferential fibers in the dorsal epidermis provide dorsoventrally oriented stiffness and a corset to canalize forces that are generated by the lateral seam cells. Generally, bundling of F-actin increases cortical stiffness, whereas disorganized networks of shorter filaments lead to cell-surface softening (reviewed in Pegoraro et al., 2017). Hence, stiffening of the dorsal and ventral molecular corset not only transmits forces from seam cells causing a circumferential squeezing of the internal tissue (Vuong-Brender et al., 2017) but also provides a mechanical barrier to guide the re-allocation of space for the internal tissues along the anteroposterior axis. Published data further supports the notion that DLG-1 might regulate this phenomenon. Some examples include (1) microinjecting increasing amounts of *dlg-1* could rescue morphogenetic phenotypes in a hypercontractility mutant [*rga-2(hd102)*; Diogon et al., 2007] and it was proposed that the elevated level of DLG-1 strengthened junctions toward extensive pulling forces exerted by actomyosin from the lateral epidermis. (2) Pathways that compensate for the loss of DLG-1 have been characterized for other tissues (Pilipiuk et al., 2009). Thus, an increase in DLG-1 might slow down elongation rate by counterbalancing tensile forces exerted from the actomyosin. (3) Downregulation of DLG-1 reduces the amount of F-actin in *C. elegans* intestinal cells through an interaction with IFO-1, a protein responsible for F-actin organization in this tissue (Carberry et al., 2012) and DLG-1 might interact similarly with actin organizers in the epidermis. For instance through VAB-9, which interacts with the CCC and anchors F-actin bundles at dorsal and ventral seam cell junctions (Simske et al., 2003; Vuong-Brender et al., 2018).

An alternative explanation for the phenotypes that we describe would be that tensile forces in seam cells are reduced, e.g., through reduced non-muscle myosin II (NMM II) activity. For that reason, we performed laser ablations on seam cell junctions and found a decrease in the plateau (final distance of junctional vertices) reached in *pha-1(e2123)* (Figure 5). This reduction became more apparent the further the embryo was elongated. It should be noted though that the initial recoil did not differ between WT and *pha-1(e2123)*. This initial recoil is the ratio of the tensile force the junction has to sustain prior to the ablation and the viscosity coefficient of the cytoplasm (Liang et al., 2016). Hence, it reflects the level of mechanical stress. As this was not significantly changed, it seems unlikely that changes in NMM II activity are the cause for the *pha-1(e2123)* phenotypes. However, other factors that have a presumably lesser influence on cortical tension like the organization of the cytoskeletal meshwork (e.g., amount and composition of actin crosslinkers) do not necessarily result in drastic changes of the initial recoil but would alter the material properties of epidermal cells, thereby underlining the notion that tissue stiffness and/or viscosity are most likely affected in *pha-1(e2123)*. We consider this the most likely reason since it can also explain the lack of body retraction in *pha-1(e2123)* (Figure 6).

Another prominent feature of embryonic arrest in *pha-1(e2123)* is the majority of individuals arresting at twofold stage (pat-phenotype). At this stage, further elongation of the embryo requires the underlying muscle tissue. Deficits in muscle development and/or function or inhibition of force transmission between muscles and epidermis lead to failure of elongation past the twofold stage (Williams and Waterston, 1994; Zhang et al., 2011). During phenotypic analyses of *pha-1(e2123)*, we discovered a delay of the onset of muscle twitching and a reduction in twitching intensities (Figures 6C,D), indicating a problem in muscle development or force transmission. This notion receives further support by our finding that downregulation of VAB-10 *via* feeding RNAi strongly increases embryonic lethality in *pha-1(e2123)* (Figure 3A) while it leads to larval lethality in the WT background. Two isoforms of VAB-10 are expressed (and both were downregulated by our RNAi), of which VAB-10A is a component of *C. elegans* hemidesmosomes that are mechanical linkers transmitting contractile forces of muscle tissue to the cuticle *via* epidermal cells (Suman et al., 2019). Therefore, loss of PHA-1 causes additional phenotypes beyond cell-autonomous epidermal morphogenesis that might again be caused by altered epidermal properties including tissue–tissue attachment.

Taken together, our work demonstrates the genetic connection between the *pha-1/sup-35* element and epithelial differentiation. It also reveals several tissue mechanical phenotypes that warrant further characterization, especially a deeper investigation of molecular interactions at the tissue, or ideally, at single cell level to be able to deconvolve such complex phenotypes as observed in *pha-1(e2123)* embryos.

## Data Availability Statement

The original contributions presented in the study are included in the article/supplementary material, further inquiries can be directed to the corresponding author.

## Author Contributions

CP conceived and supervised the project and wrote the manuscript with input from CL. CL performed the experiments. CL and CP analyzed the data. Both authors contributed to the article and approved the submitted version.

## Conflict of Interest

The authors declare that the research was conducted in the absence of any commercial or financial relationships that could be construed as a potential conflict of interest.

## Publisher’s Note

All claims expressed in this article are solely those of the authors and do not necessarily represent those of their affiliated organizations, or those of the publisher, the editors and the reviewers. Any product that may be evaluated in this article, or claim that may be made by its manufacturer, is not guaranteed or endorsed by the publisher.

## Footnotes

1
http://www.lifesciences.sourcebioscience.com/clone-products/mirna--rnai-resources.aspx
